# Ambiguity, Weakness, and Regularity in Probabilistic Büchi Automata

**DOI:** 10.1007/978-3-030-45231-5_27

**Published:** 2020-04-17

**Authors:** Christof Löding, Anton Pirogov

**Affiliations:** 8 ENS/CNRS, Université Paris-Saclay, Cachan, France; 9 University of Duisburg-Essen, Duisburg, Germany; RWTH Aachen University, Templergraben 55, 52062 Aachen, Germany

**Keywords:** probabilistic, Büchi, automata, ambiguity, weak

## Abstract

Probabilistic Büchi automata are a natural generalization of PFA to infinite words, but have been studied in-depth only rather recently and many interesting questions are still open. PBA are known to accept, in general, a class of languages that goes beyond the regular languages. In this work we extend the known classes of restricted PBA which are still regular, strongly relying on notions concerning ambiguity in classical $\omega$-automata. Furthermore, we investigate the expressivity of the not yet considered but natural class of weak PBA, and we also show that the regularity problem for weak PBA is undecidable.

## 1 Introduction

Probabilistic finite automata (PFA) are defined similarly to nondeterministic finite automata (NFA) with the difference that each transition is equipped with a probability (a value between 0 and 1), such that for each pair of state and letter, the probabilities of the corresponding outgoing transitions sum up to 1. PFA have been investigated already in the 1960ies in the seminal paper of Rabin [18]. But while the development of the theory of automata on infinite words also started around the same time [7], the model of probabilistic automata on infinite words has first been studied systematically in [3]. The central model in this theory is the one of probabilistic Büchi automata (PBA), which are syntactically the same as PFA. The acceptance condition for runs is defined as for standard nondeterministic Büchi automata (NBA): a run on an infinite word is accepting if it visits an accepting state infinitely often (see [23,24] for an introduction to the theory of automata on infinite words). In general, for probabilistic automata one distinguishes different criteria of when a word is accepted. In the positive semantics, it is required that the probability of the set of accepting runs is greater than 0, in the almost-sure semantics it has to be 1, and in the threshold semantics it has to be greater than a given value $\lambda$ between 0 and 1. It is easy to see that PFA with positive or almost-sure semantics can only accept regular languages, because these conditions correspond to the fact that there is an accepting run or

⋆ This work is supported by the German research council (DFG) Research Training Group 2236 UnRAVeL

that all runs are accepting. For infinite words the situation is different, because single runs on infinite words can have probability 0. Therefore, the existence of an accepting run is not the same as the set of accepting runs having probability greater than 0 (similarly, almost-sure semantics is not equivalent to all runs being accepting). And in fact, it turns out that PBA with positive (or almost-sure) semantics can accept non-regular languages [3]. This naturally raises the question under which conditions a PBA accepts a regular language.

In [3] a subclass of PBA that accept only regular languages (under positive semantics) is introduced, called uniform PBA. The definition uses a semantic condition on the acceptance probabilities in end components of the PBA. A syntactic class of PBA that accepts only regular languages (under positive and almost-sure semantics) are the hierarchical PBA (HPBA) introduced in [8]. The state space of HPBA is partitioned into a sequence of layers such that for each pair of state and letter there is at most one transition that does not increase the layer. Decidability and expressiveness questions for HPBA have been studied in more detail in [11,10]. While HPBA accept only regular languages for positive and almost-sure semantics, it is not very hard to come up with HPBA that accept non-regular languages under the threshold semantics [8,11] (see also the example in Figure 2(a) on page 531). Restricting HPBA further such that there are only two layers and all accepting states are on the first layer leads to a class of PBA (called simple PBA, SPBA) that accept only regular languages even under threshold semantics [9].

In this paper, we are also interested in the question under which conditions PBA accept only regular languages. We identify syntactical patterns in the transition structure of PBA whose absence guarantees regularity of the accepted language. These patterns have been used before for the classification of the degree of ambiguity of NFA and NBA [25,19,16]. The degree of ambiguity of a nondeterministic automaton corresponds to the maximal number of accepting runs that a single input word can have. For NBA, the ambiguity can (roughly) be uncountable, countable, or finite. For positive semantics, we show that PBA whose transition structure corresponds to at most countably ambiguous NBA, accept only regular languages. For almost-sure semantics, we need a slightly stronger condition for ensuring regularity. But both classes that we identify are easily seen to strictly subsume the class of HPBA. For the emptiness and universality problems for these classes we obtain the same complexities as the ones for HPBA. In the case of threshold semantics, we show that finite ambiguity is a sufficient condition for regularity of the accepted language, generalizing a corresponding result for PFA from [12]. The class of finitely ambiguous PBA strictly subsumes the class of SPBA.

Besides the relation between regularity and ambiguity in PBA, we also investigate the class of weak PBA (abbreviated PWA). In weak Büchi automata, the set of accepting states is a union of strongly connected components of the automaton. We show that PWA with almost-sure semantics define the same class of languages as PBA with almost-sure semantics (which implies that with positive semantics PWA define the same class as probabilistic co-Büchi automata).

This is in correspondence to results for non-probabilistic automata: weak automata with universal semantics (a word is accepted if all runs are accepting) define the same class as Büchi automata with universal semantics, and nondeterministic weak automata correspond to nondeterministic co-Büchi automata (see, e.g., [17], where weak automata are called weak parity automata). Furthermore, it is known that universal Büchi automata, respectively nondeterministic co-Büchi automata, can be transformed into equivalent deterministic automata (with the same acceptance condition). An analogue of deterministic automata in the probabilistic setting are the so-called 0/1 automata, in which each word is either accepted with probability 0 or with probability 1. It is known that almost-sure PBA can be transformed into equivalent 0/1 PBA (see the proof of Theorem 4.13 in [4]). Concerning weak automata, a language can be accepted by a deterministic weak automaton (DWA) if, and only if, it can be accepted by a deterministic Büchi and by a deterministic co-Büchi automaton (this follows from results in [14], see [6] for a more direct construction). We show an analogous result in the probabilistic setting: The class of languages defined by 0/1 PWA corresponds to the intersection of the two classes defined by PWA with almost-sure semantics and with positive semantics, respectively. It turns out that this class contains only regular languages, that is, 0/1 PWA define the same class as DWA.

We also show that the regularity problem for PBA is undecidable (the problem of deciding for a given PBA whether its language is regular). For PBA with positive semantics this is not surprising, as for those already the emptiness problem is undecidable [4]. However, for PBA with almost-sure semantics the emptiness and universality problems are decidable [1,2,8]. We show that regularity is undecidable already for PWA with almost-sure or with positive semantics. The proof also yields that it is undecidable for a fixed regular language whether a given PWA accepts this language.

This work is organized as follows. After introducing basic notations in Section 2 we first characterize various regular subclasses of PBA that we derive from ambiguity patterns in Section 3 and then we derive some related complexity results in Section 4. In Section 5 we present our results concerning weak probabilistic automata and in Section 6 we conclude.

## 2 Preliminaries

First we briefly review some basic definitions.

If $\Sigma$ is a finite alphabet, then $\Sigma^*$ is the set of all finite and $\Sigma^\omega$ is the set of all infinite *words* $w = w_0w_1\dots$ with $w_i \in \Sigma$. For a word $w$ we denote by $w(i)$ the $i$-th symbol $w_i$.

Classical automata used in this work have usually the shape $(Q, \Sigma, \Delta, Q_0, F)$, where $Q$ is a finite set of states, $\Sigma$ a finite alphabet, $\Delta \subseteq Q \times \Sigma \times Q$ is the transition relation and $Q_0, F \subseteq Q$ are the sets of initial and final states, respectively.

We write $\Delta(p, a) := \{q \in Q \mid (p, a, q) \in \Delta\}$ to denote the set of *successors* of $p \in Q$ on symbol $a \in \Sigma$, and $\Delta(P, w)$ for $P \subseteq Q, w \in \Sigma^*$ with the usual meaning, i.e., states reachable on word $w$ from any state in $P$.

A *run* of an automaton on a word $w \in \Sigma^\omega$ is an infinite sequence of states $q_0, q_1, \ldots$ starting in some $q_0 \in Q_0$ such that $(q_i, w(i), q_{i+1}) \in \Delta$ for all $i \geq 0$. We say that a set of runs is *separated (at time i)* when the prefixes of length $i$ of those runs are pairwise different.

As usual, an automaton is *deterministic* if $|Q_0| = 1$ and $|\Delta(p, a)| \leq 1$ for all $p \in Q, a \in \Sigma$, and *nondeterministic* otherwise. For deterministic automata we may use a transition function $\delta : Q \times \Sigma \to Q$ instead of a relation.

Probabilistic automata we consider have the shape $(Q, \Sigma, \delta, \mu_0, F)$, i.e., the transition relation is replaced by a function $\delta : Q \times \Sigma \times Q \to [0, 1]$ which for each state and symbol assigns a probability distribution on successor states (i.e. $\sum_{q \in Q} \delta(p, a, q) = 1$ for all $p \in Q, a \in \Sigma$), and $\mu_0 : Q \to [0, 1]$ with $\sum_{q \in Q} \mu_0(q) = 1$ is the initial probability distribution on states. The *support* of a distribution $\mu$ is the set $\mathsf{supp}(\mu) := \{x \mid \mu(x) > 0\}$. Similarly as above, we may write $\delta(\mu, w)$ and mean the resulting probability distribution after reading $w \in \Sigma^*$, when starting with probability distribution $\mu$.

For a probabilistic automaton $\mathcal{A}$ the *underlying automaton* $\mathcal{A}^{\triangleleft}$ is given by recovering the transition relation $\Delta := \{(p, x, q) \mid \delta(p, x, q) > 0\}$ of positively reachable states and the initial state set $Q_0 := \mathsf{supp}(\mu_0)$.

As usual, a run of an automaton for finite words is *accepting* if it ends in a final state. For automata on infinite words, run acceptance is determined by the Büchi (run visits infinitely many final states) or Co-Büchi (run visits finitely many final states) conditions.

We write $p \xrightarrow{x} q$ if there exists a path from $p$ to $q$ labelled by $x \in \Sigma^+$ and $p \to q$ if there exists some $x$ such that $p \xrightarrow{x} q$. The *strongly connected component (SCC)* of $p \in Q$ is $\mathsf{scc}(p) := \{q \in Q \mid p = q \text{ or } p \to q \text{ and } q \to p\}$. The set $\mathsf{SCCs}(\mathcal{A}) := \{\mathsf{scc}(q) \mid q \in Q\}$ is the set of all SCCs and partitions $Q$. An SCC is *accepting* (*rejecting*) if all (no) runs that stay there forever are accepting. An SCC is *useless* if no accepting run can continue from there. An automaton is *weak*, if the set of final states is a union of its SCCs. In this case, Büchi and Co-Büchi acceptance are equivalent and we treat weak automata as Büchi automata.

A classical automaton is *trim* if it has no useless SCCs, whereas a probabilistic automaton is trim if it has at most one useless SCC, which is a rejecting sink that we canonically call $q_{rej}$. We assume w.l.o.g. that all considered automata are trim, which also means that in an underlying automaton the sink $q_{rej}$ is removed.

We call transitions of probabilistic automata that have probability 1 *deterministic* and otherwise *branching.* If there are transitions $p \xrightarrow{a} q$ and $p \xrightarrow{a} q'$ with $q \neq q'$, we call this pattern a *fork.* Every branching transition clearly has at least one fork. We call a $(p, q, q')$ fork *intra-SCC*, if $p, q, q'$ are all in the same SCC, otherwise it is an *inter-SCC* fork. A run of an automaton is *deterministic* if it never goes through forks, and *limit-deterministic* if it goes only through finitely many forks. We say that two deterministic runs *merge* when they reach the same state simultaneously. For a finite run prefix $\rho$, we call all valid runs with this prefix *continuations* of $\rho$.

A classical automaton $\mathcal{A}$ *accepts* $w \in \Sigma^\omega$ if there exists an accepting run on $w$, and the language $L(\mathcal{A})$ *recognized* by $\mathcal{A}$ is the set of all accepted words. If $P$ is a set of states of an automaton, we write $L(P)$ for the language accepted by this automaton with initial state set $P$. For sets consisting of one state $q$, we write $L(q)$ instead of $L(\{q\})$.

For a probabilistic automaton $\mathcal{A}$ and an input word $w$ (finite or infinite), the transition structure of $\mathcal{A}$ induces a probability space on the set of runs of $\mathcal{A}$ on $w$ in the usual way. We do not provide the details here but rather refer the reader not familiar with these concepts to [4]. In general, we write $\mathsf{Pr}(E)$ for the probability of a measurable event $E$ in a probability space. For probabilistic automata, we consider *positive*, *almost-sure* and *threshold* semantics, i.e., an automaton accepts $w$ if the probability of the set of accepting runs on $w$ is $> 0$, $=1$ or $>\lambda$ (for some fixed $\lambda \in ]0,1[$), respectively. For an automaton $\mathcal{A}$ these languages are denoted by $L^{>0}(\mathcal{A}), L^{=1}(\mathcal{A})$ and $L^{>\lambda}(\mathcal{A})$, respectively, whereas $L(\mathcal{A}) := L(\mathcal{A}^{\triangleleft})$ is the language of the underlying automaton. A probabilistic automaton is $0/1$ if all words are accepted with either probability 0 or 1 (in this case the languages with the different probabilistic semantics coincide).

To denote the type of an automaton, we use abbreviations of the form XYA$^{(\gamma)}$ where the type of transition structure is denoted by X $\in$ { D (det.), N (nondet.), P (prob.) }, the acceptance condition is specified by Y $\in$ { F (finite word), B (Büchi), C (Co-Büchi), W (Weak) }, and for probabilistic transitions the semantics for acceptance is given by $\gamma \in \{>0,=1,>\lambda, 0/1\}$.

By $\mathbb{L}^{(\gamma)}(\mathsf{XYA})$ we denote the whole class of languages accepted by the corresponding type of automaton. If $\mathbb{L}$ is a set of languages, then $\overline{\mathbb{L}}$ denotes the set of all complement languages (similarly, for a language $L$, we denote by $\overline{L}$ its complement), and $\mathsf{BCl}(\mathbb{L})$ the set of all finite boolean combinations of languages in $\mathbb{L}$. We use the notion of *regular language* for finite words and for infinite words (the type of words is always clear from the context).

## 3 Ambiguity of PBA

Ambiguity of automata refers to the number of different accepting runs on a word or on all words. An automaton is *finitely ambiguous* (on $w$) if there are at most $k$ different accepting runs (on $w$) for some fixed $k \in \mathbb{N}$, and in case of at most one accepting run it is called *unambiguous*. If on each word there are only finitely many accepting runs, but no constant upper bound over all words, then it is *polynomially ambiguous* if the number of different run prefixes that are possible for any word prefix of length $n$ can be bounded by a polynomial in $n$, and otherwise *exponentially ambiguous*. Finally, if if there exist words that have infinitely many runs, but no word on which there are uncountably many accepting runs, then it is *countably ambiguous*, and otherwise it is *uncountably ambiguous*.

In [16] (see also [19]), a syntactic characterization of those classes is presented for NBA by simple patterns of states and transitions. We define those patterns here and refer to [16] for further details. An automaton $\mathcal{A}$ has an *IDA pattern*

if there exist two states $p \neq q$ and a word $v \in \Sigma^*$ such that $p \xrightarrow{v} p$, $p \xrightarrow{v} q$ and $q \xrightarrow{v} q$. If additionally $q \in F$, then this is also an $\mathrm{IDA}_F$ pattern. Finally, $\mathcal{A}$ has an *EDA pattern* if there exists a state $p$ and $v \in \Sigma^*$ such that there are two different paths $p \xrightarrow{v} p$, and if additionally $p \in F$, this is also an $\mathrm{EDA}_F$ pattern. If a PBA has no EDA pattern, we call it *flat*, reflecting the naming of a similar concept in other kinds of transition systems (e.g. [15]). The names IDA and EDA abbreviate "infinite/exponential degree of ambiguity", which they indicated in the original NFA setting, and we keep those names for consistency.

By $k$-NBA, $n^k$-NBA, $2^n$-NBA, $\aleph_0$-NBA we denote the subsets of at most finitely, polynomially, exponentially and countably ambiguous NBA (and similarly for other types of automata). When speaking about ambiguity of some PBA $\mathcal{A}$, we mean the ambiguity of the trimmed underlying NBA $\mathcal{A}^{\triangleleft}$.

In [8], hierarchical PBA (HPBA) were identified as a syntactic restriction on PBA which ensures regularity under positive and almost-sure semantics. A PBA with a unique initial state is hierarchical, if it admits a ranking on the states such that at most one successor on a symbol has the same rank, and no successor has a smaller rank. A HPBA has $k$ levels if it can be ranked with only $k$ different values. Simple PBA (SPBA) were introduced in [9] and are restricted HPBA with two levels such that all accepting states are on level 0.

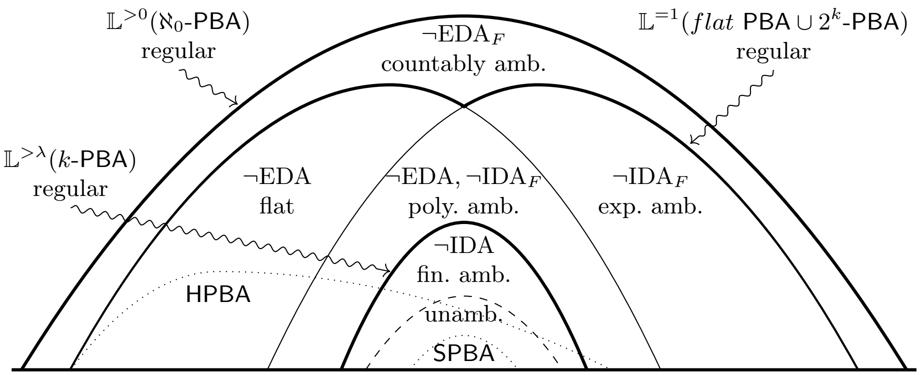

Fig. 1: Illustration of the automata classes with restricted ambiguity as presented for NBA in [16], which are characterized by the absence of the state patterns $\mathrm{IDA}, \mathrm{IDA}_F, \mathrm{EDA}$, and $\mathrm{EDA}_F$ and their relation to the restricted classes called "Hierarchical PBA" (HPBA) [8] and "Simple PBA" (SPBA) [9]. We identify classes in this hierarchy which can be seen as extensions "in spirit" of respectively SPBA and HPBA, subsuming them while also preserving their good properties, as e.g. definition by syntactic means, regularity under different semantics and several complexity results.

First, we show how HPBA relate to the ambiguity hierarchy, which can easily be derived by inspection of the definitions. A visual illustration is given in Figure 1.

**Proposition 1 (Relation of HPBA and the ambiguity hierarchy).**

1. HPBA $\subset$ *flat PBA* $\subset$ $\aleph_0$-PBA.
2. $k$-PBA $\not\subseteq$ HPBA *and* HPBA $\not\subseteq$ $k$-PBA.
3. SPBA $\subset$ *unambiguous PBA* $\subset$ $k$-PBA.

Starting from these observations, this work was motivated by the question whether the ambiguity restrictions, which were only implicit in HPBA and SPBA, can be used explicitly to get larger classes with good properties. In the following we will positively answer this question.

### 3.1 From classical to probabilistic automata

First, we observe that probabilistic automata can recognize regular languages even under severe ambiguity restrictions.

**Proposition 2.** *Let $\mathcal{A}$ be a DBA. Then there exists an unambiguous PBA $\mathcal{B}$ such that $L^{>0}(\mathcal{B}) = L^{=1}(\mathcal{B}) = L(\mathcal{A})$.*

*Proof.* As $\mathcal{A}$ is a (w.l.o.g. complete) DBA, there exists exactly one run on each word and all transitions when seen as PBA must have probability 1. Clearly this unique natural 0/1 PBA obtained from $\mathcal{A}$ accepts the same language under both probable and almost-sure semantics and it is trivially unambiguous. □

Limit-deterministic NBA (LDBA) are NBA which are deterministic in all non-rejecting SCCs. The natural mapping of LDBA into PBA [4, Lemma 4.2] already trivially yields countably ambiguous automata (because the deterministic part of the LDBA cannot contain an $\mathrm{EDA}_F$ pattern, which implies uncountable ambiguity [16]). The following result shows that already unambiguous PBA under positive semantics suffice for all regular languages.

**Theorem 1.** *Let $L \subseteq \Sigma^\omega$ be a regular language.*
*Then there exists an unambiguous PBA $\mathcal{B}$ such that $L^{>0}(\mathcal{B}) = L$.*

*Proof (sketch).* Let $\mathcal{A} = (Q, \Sigma, \delta, q_0, c)$ be a deterministic parity automaton accepting $L$, i.e., a finite automaton with priority function $c : Q \to \{1, \dots, m\}$ such that $w \in L(\mathcal{A})$ iff the smallest priority assigned to a state on the unique run of $\mathcal{A}$ on $w$ which is seen infinitely often is even.

We construct an unambiguous LDBA for $L$, which then easily yields a $\mathrm{PBA}^{>0}$ by assigning arbitrary probabilities ([4, Lemma 4.2]) without influencing the ambiguity. If the parity automaton $\mathcal{A}$ has $m$ priorities, the LDBA $\mathcal{B}$ can be obtained by taking $m+1$ copies, where $m$ of them are responsible for one priority each, and one is modified to guess which priority $i$ on the input word is the most important one appearing infinitely often along the run of $\mathcal{A}$, and correspondingly switch into the correct copy. This switching is done unambiguously for the first position after which no priority more important than $i$ appears. □

### 3.2 From probabilistic to classical automata

First we establish a result for flat PBA, i.e. PBA that have no EDA pattern. In automata without EDA pattern there are no states which are part of two different cycles labeled by the same finite word. Even though we defined flat PBA by using an ambiguity pattern, the set of flat PBA does not correspond to an ambiguity class, but it is useful for our purposes due to the following property:

**Lemma 1.** *If $\mathcal{A}$ is a flat PBA and $w \in \Sigma^\omega$, then the probability of a run of $\mathcal{A}$ on $w$ to be limit-deterministic is 1.*

*Proof.* Let $\mathsf{Runs}(\mathcal{A}, w)$ denote the set of all runs of $\mathcal{A}$ on $w$ and $\mathsf{nldRuns}(\mathcal{A}, w)$ denote the subset containing all such runs that are not limit-deterministic. As $\mathcal{A}$ is flat, it has no EDA and thus also no $\mathrm{EDA}_F$ pattern, hence $\mathcal{A}$ is at most countably ambiguous (by [16]). Moreover, there are not only at most countably many accepting runs on any word, but also countably many rejecting runs (which can be seen by a simple generalization of [16, Lemma 4]). But as all runs are disjoint events, each run $\rho$ that uses infinitely many forks has probability 0, and the total number of runs is countable, we can see that

$$\mathsf{Pr}(\mathsf{Runs}(\mathcal{A}, w) \setminus \mathsf{nldRuns}(\mathcal{A}, w)) = \sum_{\rho \in \mathsf{Runs}(\mathcal{A}, w)} \mathsf{Pr}(\rho) \quad - \sum_{\rho \in \mathsf{nldRuns}(\mathcal{A}, w)} \mathsf{Pr}(\rho) = 1 - 0 = 1. \qquad \square$$

The following lemma characterizes acceptance of PBA under extremal semantics with restricted ambiguity and is crucial for the constructions in the following sections:

**Lemma 2 (Characterizations for extremal semantics).**
*Let $\mathcal{A}$ be a PBA.*

1. *If $\mathcal{A}$ is at most countably ambiguous, then* $w \in L^{>0}(\mathcal{A}) \Leftrightarrow$ *there exists an accepting run on $w$ that is limit-deterministic.*
2. *If there are finitely many accepting runs of $\mathcal{A}$ on $w$, then* $w \in L^{=1}(\mathcal{A}) \Leftrightarrow$ *all runs on $w$ are accepting and limit-deterministic.*
3. *If $\mathcal{A}$ is flat, then* $w \in L^{=1}(\mathcal{A}) \Leftrightarrow$ *there is no limit-deterministic rejecting run on $w$.*

*Proof.* (1.) : For contradiction, assume that every accepting run on $w$ goes through forks infinitely often. But then the probability of every individual accepting run on $w$ is 0. Each run is a measurable event (it is a countable intersection of finite prefixes) and clearly disjoint from other runs, as two different runs must eventually differ after a finite prefix. But as the number of accepting runs is countable by assumption, by $\sigma$-additivity it follows that the probability of all accepting runs is also 0, contradicting the fact that $w \in L^{>0}(\mathcal{A})$.

For the other direction, pick a limit-deterministic accepting run $\rho$ of $\mathcal{A}$ on $w$ and let $uv = w$ and $q \in Q$ such that the state of $\rho$ after reading $u$ is $q$ and there are no forks visited on $v$. Clearly, the probability to be in $q$ after $u$ in a run of $\mathcal{A}$ is positive (because $u$ is finite), and the probability that $\mathcal{A}$ continues like $\rho$ from $q$ on $v$ is 1. Hence, the probability of $\rho$ is positive.

(2.) : The ($\Leftarrow$) direction is obvious. We now proceed to show ($\Rightarrow$). Take some time $t$ after which all accepting runs on $w$ separated. Assume that some accepting run $\rho$ is not limit-deterministic. But then $\rho$ goes through infinitely many forks after $t$ which with positive probability lead to a successor from which the probability to accept is 0, and the probability of following $\rho$ is also 0. As the probability to follow $\rho$ until time $t$ is positive, but after that the probability to accept is 0, this implies that there is a positive probability that $\mathcal{A}$ rejects $w$. Therefore, all accepting runs on $w$ must be limit-deterministic. Now assume that some run $\rho$ on $w$ is rejecting. Following this run until the time at which $\rho$ is separated from all accepting runs has positive probability and all continuations must be also rejecting, so $\mathcal{A}$ must reject $w$.

(3.) : Clearly ($\Rightarrow$) holds, because a limit-deterministic rejecting run has positive probability, i.e., if such a run exists on $w$, then $\mathcal{A}$ cannot accept almost surely. For ($\Leftarrow$), observe that because $\mathcal{A}$ is flat, we know by Lemma 1 that with probability 1 runs are limit-deterministic. Hence, if there exists no limit-deterministic rejecting run on $w$ (which would have positive probability), then with probability 1 runs are limit-deterministic and accepting. □

Using these characterizations, we can provide simple constructions from probabilistic to classical automata.

**Theorem 2.** *Let $\mathcal{A}$ be a PBA that is at most countably ambiguous. Then $L^{>0}(\mathcal{A})$ is a regular language.*

*Proof (sketch).* An NBA construction taking two copies of the PBA, where in the first copy no state is accepting and the second copy has no forks, with the purpose of guessing a limit-deterministic accepting run. □

**Corollary 1.** *If $L^{>0}(\mathcal{A})$ is not regular, then it contains an* EDA$_F$ *pattern.*

**Theorem 3.** *Let $\mathcal{A}$ be a PBA that is at most exponentially ambiguous or flat. Then $L^{=1}(\mathcal{A})$ is regular and recognizable by DBA.*

*Proof (sketch).* Both cases (exp. ambiguous or flat) shown using a deterministic breakpoint construction resulting in a DBA. In one case it checks whether all runs are accepting, in the other it checks that there are no limit-deterministic rejecting runs. □

**Corollary 2.** *If $L^{=1}(\mathcal{A})$ is not regular, then $\mathcal{A}$ contains both an* EDA *and an* IDA$_F$ *pattern.*

The corollaries above follow directly from the theorems and the syntactic characterization of ambiguity classes [16]. The following proposition states that these characterizations of regularity in terms of the ambiguity patterns are tight.

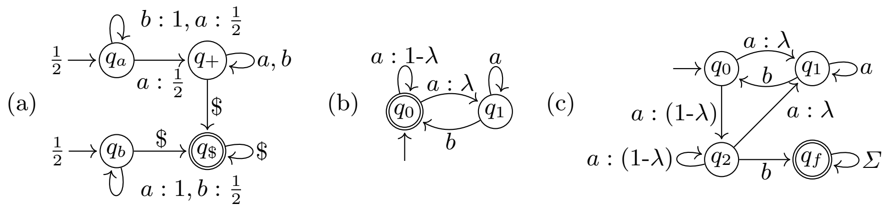

Fig. 2: (a) Some PWA which accepts the non-regular language $\{ w = (a+b)^*\$^\omega \mid \#_a(w) > \#_b(w) \}$ with a threshold of $\frac{1}{2}$, where $\#_x(w)$ denotes the number of occurrences of $x \in \Sigma$ in $w \in \Sigma^\omega$. (b) A family of PBA $\mathcal{P}_\lambda$ from [4] such that $\mathbb{L}^{>0}(\mathcal{P}_\lambda)$ is not regular for any $\lambda \in \mathbb{R}$. (c) A family of PWA $\tilde{\mathcal{P}}_\lambda$ (closely related to [4, Fig. 6]) such that $\mathbb{L}^{=1}(\tilde{\mathcal{P}}_\lambda)$ is not regular for any $\lambda \in \mathbb{R}$.

**Proposition 3.** *There exist PBA...*

1. *...with* $\mathrm{EDA}_F$ *pattern (i.e. uncountably ambiguous) that accept non-regular languages under positive semantics.*
2. *...with no* $\mathrm{EDA}_F$ *pattern (i.e. countably ambiguous) that accept non-regular languages under almost-sure semantics.*

*Proof.* (1.) Note that this statement just means that there are PBA accepting non-regular languages, which is well known. For example, the automata family from [4, Fig. 3], depicted in Figure 2(b), accepts non-regular languages under positive semantics and clearly contains an $\mathrm{EDA}_F$ pattern, e.g. there are two different paths from $p_0$ to $p_0$ on the word *aab*.

(2.) The automata family depicted in Figure 2(c) is a simple modification of the PBA family depicted in [4, Fig. 6] and recognizes the same non-regular languages under almost-sure semantics. It does not contain an $\mathrm{EDA}_F$ pattern, because the accepting state is a sink, but it does contain an $\mathrm{IDA}_F$ and an EDA pattern (both e.g. on *aab*), so it is countably ambiguous and not flat. □

This completes our classification of regular subclasses of PBA under extremal semantics that are defined by ambiguity patterns, showing that going beyond the restricted classes presented above (by allowing more patterns) in general leads to a loss of regularity.

Notice that the presented constructions do not track exact probabilities, just whether transitions have a probability $> 0$ or $= 1$. This is a noteworthy observation, as in general, the probabilities do matter for PBA, as shown in [4, Thm. 4.7, Thm. 4.11].

**Proposition 4.** *Let $\mathcal{A}$ be a PBA. The exact probabilities in $\mathcal{A}$ do not influence $L^{>0}(\mathcal{A})$ if $\mathcal{A}$ is at most countably ambiguous, and $L^{=1}(\mathcal{A})$ if $\mathcal{A}$ is at most exponentially ambiguous or flat.*

### 3.3 Threshold Semantics

In this section we consider PBA under threshold semantics and we will see that in this setting, we lose regularity much earlier than in the case of extremal semantics, but there is still the large and natural subclass of finitely ambiguous PBA that retains regularity. Before we can show this, we need to derive a suitable characterization of such languages.

We derive it from the following simple observation, which was also used more implicitly in the proof that Simple HPBA with threshold semantics are equivalent to DBA in [9].

**Lemma 3.** *Let $\mathcal{A}$ be a PBA. Then for every threshold $\lambda \in ]0,1]$, there exists a finite set of probability values $V_{\geq\lambda} \subset [\lambda, 1]$ such that for every finite run prefix with probability $v$ in $\mathcal{A}$ we have $v \geq \lambda \Rightarrow v \in V_{\geq\lambda}$.*

*Proof.* Observe that given a finite set of real numbers $R \subset [0,1]$, the set $R_{\geq\lambda} := \{r \mid r = \prod_i r_i \geq \lambda,\ r_i \in R\}$ must be finite, as in any sequence $p_1 p_2 \ldots$ of $p_i \in R$, only at most $m = \lceil \log_\lambda(\max R) \rceil$ values can be $< 1$ and such that the product of the sequence remains $\geq \lambda$. In our case, let $R$ be the set of distinct probabilities assigned to edges (including the initial edges) in $\mathcal{A}$. As every finite run prefix by definition has the probability given by the product of the edge probabilities, this implies the statement. □

If there is just one accepting run (i.e., the automaton is unambiguous), one can easily construct a nondeterministic automaton that guesses an accepting run and tracks it along with its probability value, of which there are only finitely many above the threshold. In the case that there are multiple accepting runs, for acceptance only the sum of their probabilities matters. As individual runs can in principle have arbitrarily small probability values, it is not obvious that the same approach (tracking a set of runs) can work. Determining a suitable cut-off point is not as simple, because it is not apparent when a single run becomes so improbable that it does not matter among the others. However, we will now show that such a cut-off point must exist:

**Lemma 4.** *Let $\mathcal{A}$ be a PBA, $\lambda \in ]0,1]$ a threshold and $k \in \mathbb{N}$. There exists $\varepsilon_k \in ]0, \lambda]$ such that for all sets $R^t = \{\rho_i^t\}_{i=1}^j$ of at most $j \leq k$ different run prefixes in $\mathcal{A}$ of the same length $t \in \mathbb{N}$, $\mathsf{Pr}(R^t) = \sum_{i=1}^j \mathsf{Pr}(\rho_i^t) < \lambda$ implies that $\mathsf{Pr}(R^t) < \lambda - \varepsilon_k$.*

*Proof.* We prove this by induction on the number of runs $k$. For $k = 1$, i.e. a single run prefix, let $V_{\geq\lambda}$ be the finite (by Lemma 3) set of different probability values $\geq \lambda$ and let $E$ be the set of distinct probabilities in the automaton $\mathcal{A}$. Then clearly $v_{\max,<\lambda} := \max\{a \cdot b \mid a \cdot b < \lambda, a \in V_{\geq\lambda}, b \in E\}$ is the largest probability value $< \lambda$ that can correspond to a finite run prefix in $\mathcal{A}$. Hence, we can just pick an $\varepsilon_1 < \lambda - v_{\max,<\lambda}$ and immediately get that for any run prefix with probability $v < \lambda$, we have that $v \leq v_{\max,<\lambda} < \lambda - \varepsilon_1$.

Now assume the statement holds for all sets with at most $k$ run prefixes. Let $R^t$ be a set of $k+1$ of different run prefixes of the same length such that

$\mathsf{Pr}(R^t) < \lambda$ and let $\varepsilon := \varepsilon_k$. Then we know that for every subset $S$ of at most $k$ runs of $R^t$ we have $\mathsf{Pr}(S) < \lambda - \varepsilon$. Also, every single run prefix can by Lemma 3 have one of only finitely many probability values in $V_{\geq\varepsilon}$ that are $\geq \varepsilon$ and there exists a value $v_{\max,<\varepsilon}$ denoting the largest possible probability value $< \varepsilon$ that a single run prefix can have.

If there exists a run prefix $\rho \in R^t$ with probability value $v < \varepsilon$, then we know that $\mathsf{Pr}(R^t) = \mathsf{Pr}(R^t \setminus \{\rho\}) + v < (\lambda - \varepsilon) + v_{\max,<\varepsilon} < \lambda$. If every run in $R^t$ has a probability value $\geq \varepsilon$, then every run prefix in $R^t$ has as probability one of the values in $V_{\geq\varepsilon}$. Consider all sums of $k$ values from $V_{\geq\varepsilon}$, which are finitely many, and pick the largest sum $s$ which is $< \lambda$. Choose $\varepsilon_{k+1}$ such that $\varepsilon_{k+1} < \min(\varepsilon - v_{\max,<\varepsilon}, \lambda - s)$ to account for both cases. □

From this we can derive the following characterization of languages accepted by finitely ambiguous PBA under threshold semantics:

**Lemma 5.** *Let $\mathcal{A}$ be a $k$-ambiguous PBA and $\lambda \in ]0,1]$ a threshold. There exists an $\varepsilon \in ]0, \lambda]$ such that for all $w \in \Sigma^\omega$: $w \in L^{>\lambda}(\mathcal{A})$ iff there exists a set $R$ of limit-deterministic accepting runs of $\mathcal{A}$ on $w$ with $\mathsf{Pr}(R) > \lambda$, $\mathsf{Pr}(S) \leq \lambda$ for all $S \subset R$ and at most one run $\rho \in R$ with $\mathsf{Pr}(\rho) < \varepsilon$.*

*Proof.* Clearly ($\Leftarrow$) holds, as then $w$ is accepted with probability $\geq \mathsf{Pr}(R) > \lambda$. We now show ($\Rightarrow$). In a finitely ambiguous PBA there are only finitely many different accepting runs on each word. Furthermore, as after finite time all accepting runs have separated and each accepting run that visits forks infinitely often has probability 0, accepting runs that visit forks infinitely often do not contribute positively to the acceptance probability and thus can be ignored. Hence, if $w \in L^{>\lambda}(\mathcal{A})$, there is a number of accepting runs that eventually all become deterministic and each such run has a positive probability, which must in total be $> \lambda$.

Let $R$ be a set of different limit-deterministic accepting runs of $\mathcal{A}$ on $w$ such that $\mathsf{Pr}(R) > \lambda$ and $\mathsf{Pr}(S) \leq \lambda$ for all $S \subset R$. As there are only finitely many accepting runs, such a set $R$ must exist. Furthermore, notice that each limit-deterministic run has a finite prefix which has the same probability as the whole run, so there exists a time $t$ such that the probability of the set of all different prefixes of runs in $R$ of length $t$ is exactly $\mathsf{Pr}(R)$, so that Lemma 4 applies.

Now pick an $\varepsilon := \varepsilon_k$ given by Lemma 4. We claim that at most one run $\rho \in R$ can have a probability less than $\varepsilon$. If there is no such run in $R$, we are done. Otherwise let $\rho$ be a run with $\mathsf{Pr}(\rho) =: p < \varepsilon$ and notice that by choice of $R$, we have that $\mathsf{Pr}(R \setminus \{\rho\}) =: s \leq \lambda$. It cannot be the case that $s < \lambda$, as then by Lemma 4 we have $s < \lambda - \varepsilon$, which implies that $\mathsf{Pr}(R) = s + p < \lambda$, which is a contradiction. Hence, now assume that $s = \lambda$. But then, if there is any $\rho' \neq \rho \in R$ such that $\mathsf{Pr}(\rho') =: p' < \varepsilon$, by the same argument we get the contradiction that $s - p' < \lambda - \varepsilon$ and hence $s < \lambda$. Therefore, no other run in $R$ can have a probability $< \varepsilon$. □

Now we can perform the intended automaton construction to show:

**Theorem 4.** *$L^{>\lambda}(\mathcal{A})$ is regular for each $k$-ambiguous PBA $\mathcal{A}$ and $\lambda \in ]0, 1[$.*

*Proof (sketch).* We use the characterization of Lemma 5 to construct a generalized Büchi automaton accepting $L^{>\lambda}(\mathcal{A})$. Intuitively, the new automaton just guesses at most $k$ different runs of $\mathcal{A}$ and verifies that the guessed runs are limit-deterministic and accepting. The automaton additionally tracks the probability of the runs over time, to determine whether the individual runs and their sum have enough "weight". The automaton rejects when the total probability of the guessed runs is $\leq \lambda$, one of the runs goes into the rejecting sink $q_{rej}$ or a run does not see accepting states infinitely often.

By Lemma 5 we only need to consider sets of runs with at most one run that has a probability $< \varepsilon$, where $\varepsilon := \varepsilon_k$ is given by Lemma 4. For this single run we also do not need to track the exact probability value, as its only purpose is to witness that the acceptance probability is strictly greater than $\lambda$, whereas all other runs must have one of the finitely many different probabilities which are $\geq \varepsilon$ and must sum to $\lambda$. □

This generalizes the corresponding result for PFA [12, Theorem 3]. The proof in [12] uses similar concepts, though a rather different presentation. In the setting of infinite words we additionally have to deal with a single run that has arbitrarily low probability, and we have to ensure that this probability remains positive.

After seeing that finitely ambiguous PBA retain regularity, we show that this is the best we can do under threshold semantics:

**Corollary 3.** *There are polynomially ambiguous PBA $\mathcal{A}$, that is, with an* IDA *pattern and no* EDA, $\text{IDA}_F$ *patterns, such that $L^{>\lambda}(\mathcal{A})$ is not regular even for rational thresholds $\lambda \in ]0,1[$.*

*Proof.* Follows from the fact that the PWA $\mathcal{A}$ from Figure 2(a), which recognizes a non-regular language (and is used to show Proposition 6), has just an IDA pattern in the underlying NBA, but no EDA or $\text{IDA}_F$ patterns. □

This completes our characterization of languages which are recognized by PBA that are restricted by forbidden ambiguity patterns, so that we can state our main result of this section (see Figure 1 for a visualization):

**Theorem 5.** *The following results hold about PBA with restricted ambiguity:*

- $\mathbb{L}^{>0}(k\text{-}\mathsf{PBA}) = \mathbb{L}^{>0}(\aleph_0\text{-}\mathsf{PBA}) = \mathbb{L}(\mathsf{NBA})$
- $\mathbb{L}^{=1}(k\text{-}\mathsf{PBA}) = \mathbb{L}^{=1}(2^k\text{-}\mathsf{PBA}) = \mathbb{L}^{=1}(\textit{flat}\ \mathsf{PBA}) = \mathbb{L}(\mathsf{DBA}) \subset \mathbb{L}^{=1}(\aleph_0\text{-}\mathsf{PBA})$
- $\mathbb{L}^{>\lambda}(k\text{-}\mathsf{PBA}) = \mathbb{L}(\mathsf{NBA}) \subset \mathbb{L}^{>\lambda}(n^k\text{-}\mathsf{PBA})$

*Proof.* The statements follow from the following inclusion chains:

$$\mathbb{L}(\mathsf{NBA}) \overset{(1.)}{\subseteq} \mathbb{L}^{>0}(k\text{-}\mathsf{PBA}) \overset{def.}{\subseteq} \mathbb{L}^{>0}(\aleph_0\text{-}\mathsf{PBA}) \overset{(2.)}{\subseteq} \mathbb{L}(\mathsf{NBA})$$

$$\mathbb{L}(\mathsf{DBA}) \overset{(3.)}{\subseteq} \mathbb{L}^{=1}(k\text{-}\mathsf{PBA}) \overset{def.}{\subseteq} \mathbb{L}^{=1}(2^k\text{-}\mathsf{PBA} \cup \text{flat PBA}) \overset{(4.)}{\subseteq} \mathbb{L}(\mathsf{DBA}) \overset{(5.)}{\subset} \mathbb{L}^{=1}(\aleph_0\text{-}\mathsf{PBA})$$

$$\mathbb{L}(\mathsf{NBA}) \overset{(1.)}{\subseteq} \mathbb{L}^{>0}(k\text{-}\mathsf{PBA}) \overset{(6.)}{\subseteq} \mathbb{L}^{>\lambda}(k\text{-}\mathsf{PBA}) \overset{(7.)}{\subseteq} \mathbb{L}(\mathsf{NBA}) \overset{(8.)}{\subset} \mathbb{L}^{>\lambda}(n^k\text{-}\mathsf{PBA})$$

Where the marked relationships hold due to: (1.) Theorem 1, (2.) Theorem 2, (3.) Proposition 2, (4.) Theorem 3, (5.) Proposition 3, (6.) Simple transformation by adding a new accepting sink $q_{acc}$ and modifying the initial distribution $\mu_0$ [4, Lemma 4.16], (7.) Theorem 4, (8.) Corollary 3, and (def.) by definition of the ambiguity-restricted automata classes. □

## 4 Complexity results

In this section, we state some upper and lower bounds on the complexity for deciding emptiness and universality for PBA with restricted ambiguity, derived from the characterizations and constructions presented above.

**Theorem 6.**

1. *the emptiness problem for* $\aleph_0$*-PBA*$^{>0}$ *is in* NL
2. *the universality problem for* $\aleph_0$*-PBA*$^{>0}$ *is in* PSPACE
3. *the universality problem for at most exp. ambiguous or flat PBA*$^{=1}$ *is in* NL

*Proof.* (1. + 2.) : By Theorem 2 the languages of $\aleph_0$-PBA$^{>0}$ are regular. The construction of an NBA just uses two copies of the given PBA. For emptiness, it thus suffices to guess an accepted ultimately periodic word and verify that it is accepted by the NBA, which can be done in NL. Since universality for NBA in in PSPACE [21], we also obtain (2.).

(3.): If the automaton is at most exponentially ambiguous, there are only finitely many accepting runs on each word and as we know by Lemma 2 that $w \in L^{=1}(\mathcal{A})$ iff all runs are accepting, it suffices to guess a rejecting run in $\mathcal{A}^{\lhd}$, which implies that the ultimately periodic word $w$ labelling that run can not be in $L^{=1}(\mathcal{A})$. If the automaton is flat, then we know that for each rejected word there must exist a limit-deterministic rejecting run in the underlying NBA, which we also can guess. □

| Type | regular? $>0$ | $=1$ | $>\lambda$ | Emptiness $>0$ | Emptiness $=1$ | Universality $>0$ | Universality $=1$ |
|---|---|---|---|---|---|---|---|
| $k$-PBA | ✓ | ✓ | ✓ | ∈ NL | ∈ PSPACE | ∈ PSPACE | ∈ NL |
| $n^k$-PBA | ✓ | ✓ | ✗ | ∈ NL | ∈ PSPACE | ∈ PSPACE | ∈ NL |
| $2^n$-PBA | ✓ | ✓ | ✗ | ∈ NL | ∈ PSPACE | ∈ PSPACE | ∈ NL |
| flat PBA | ✓ | ✓ | ✗ | ∈ NL c. | ∈ PSPACE c. | ∈ PSPACE c. | ∈ NL c. |
| $\aleph_0$-PBA | ✓ | ✗ | ✗ | ∈ NL c. | ∈ PSPACE c. | ∈ PSPACE c. | ∈ PSPACE |

Table 1: Summary of main results from Theorems 5 and 6 concerning PBA with ambiguity restrictions. The completeness results follow from the hardness results for HPBA (which are subsumed by flat PBA) from [8, Section 5], the PSPACE inclusion of universality for almost-sure $\aleph_0$-PBA follows from [8, Theorem 4.4].

Observe that $\aleph_0$-PBA$^{>0}$ subsume HPBA$^{>0}$ and the union of flat PBA$^{=1}$ and exp. ambiguous PBA$^{=1}$ subsumes HPBA$^{=1}$, while preserving the same complexity of the emptiness and universality problems. A summary of the main results from Theorem 5 and Theorem 6 is presented in Table 1.

We conclude with an observation relevant to the question about feasibility of PBA with restricted ambiguity for the purpose of application in e.g. model-checking or synthesis.

**Proposition 5 (Relationship to classical formalisms).**

- *There is a doubly-exponential lower bound for translation from LTL formula to countably ambiguous PBA with positive semantics.*
- *There is an exponential lower bound for conversion from NBA to countably ambiguous PBA with positive semantics.*

*Proof.* It is known [20, Theorem 2] that there is a doubly-exponential lower bound from LTL to LDBA. It is also known that LTL to NBA has an exponential lower bound (e.g. [5, Theorem 5.42]), which implies an exponential lower bound from NBA to LDBA.

By Theorem 2 there is a polynomial transformation from countably ambiguous PBA with positive semantics into LDBA, which together with the aforementioned bounds implies the claimed lower bounds. □

## 5 Weakness in Probabilistic Büchi Automata

In this section we investigate the class of probabilistic weak automata (PWA), establishing the relation between different classes defined by PWA as shown in Figure 3 (see also the description of our contribution in the introduction).

As a first remark, notice that PWA can be "complemented" by inverting accepting and rejecting states and switching between dual semantics, e.g., for a PWA $\mathcal{A}$ we have $\overline{L^{>0}(\mathcal{A})} = L^{=1}(\overline{\mathcal{A}})$, where $\overline{\mathcal{A}}$ is just $\mathcal{A}$ with inverted accepting state set $F' = Q \setminus F$.

Since the overarching theme of this paper is trying to find regular subclasses of PBA, we will next establish the following result, showing that there is no hope to find a complete syntactical characterization of regularity in PBA:

**Theorem 7.** *The regularity of PWA (and therefore of PBA) under positive, almost-sure and threshold semantics is an undecidable problem.*

*Proof (sketch).* Since $\mathbb{L}^{>\lambda}(\text{PWA}) \supseteq \mathbb{L}^{>0}(\text{PWA})$ (see Theorem 10), $\overline{\mathbb{L}^{>0}(\text{PWA})} = \overline{\mathbb{L}^{=1}(\text{PWA})}$, and the class of regular $\omega$-languages is closed under complement, it suffices to show the statement for $\text{PWA}^{=1}$. We do this by reduction from the value 1 problem for PFA, which is the question whether for each $\varepsilon > 0$ there exists a word accepted by the PFA with probability $> 1-\varepsilon$. This problem is known to be undecidable [13]. We consider a slightly modified version of the problem by assuming that no word is accepted with probability 1 by the given PFA. The problem remains undecidable under this assumption, because one can check if a PFA accepts a finite word with probability 1 by a simple subset construction.

Given some PFA $\mathcal{A}$, we construct a $\text{PWA}^{=1}$ $\mathcal{B}$ by taking a copy of $\mathcal{A}$ and extending it with a new symbol # such that from accepting states of $\mathcal{A}$ the automaton is "restarted" on #, while from non-accepting states # leads into a

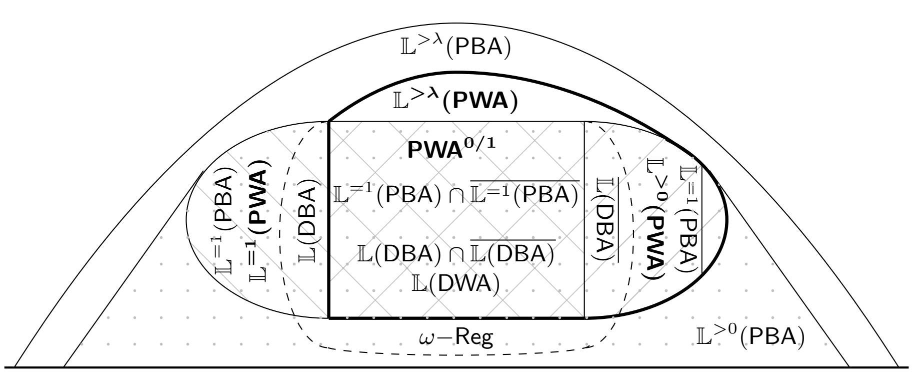

Fig. 3: Illustration of relationships between the class of languages accepted by weak probabilistic automata under various semantics with other already known classes. The overlapping patterns indicate intersection of classes, where dots mark $\mathbb{L}^{>0}(\mathsf{PBA})$, and different diagonal lines respectively $\mathbb{L}^{=1}(\mathsf{PBA})$ and $\overline{\mathbb{L}^{=1}(\mathsf{PBA})}$. The dashed line indicates intersections with different subclasses of regular languages. The class $\mathbb{L}^{>\lambda}(\mathsf{PBA})$ contains all the other depicted classes, $\mathbb{L}^{>\lambda}(\mathsf{PWA})$ contains the area inside the thick line. The depicted fact that $\mathbb{L}^{>0}(\mathsf{PWA}) = \mathbb{L}^{>\lambda}(\mathsf{PWA}) \cap \mathbb{L}^{>0}(\mathsf{PBA})$ is a conjecture, one direction is shown in Theorem 10.

new part which ensures that infinitely many # are seen and contains the only accepting state of $\mathcal{B}$. We show that $L^{=1}(\mathcal{B}) = (\Sigma^*\#)^\omega \setminus R$, where $R = \emptyset$ if $\mathcal{A}$ does not have value 1, and $R$ is non-empty but does not contain an ultimately periodic word, otherwise. This implies that $L^{=1}(\mathcal{B})$ is regular iff $\mathcal{A}$ does not have value 1. □

We will now show that PWA with almost-sure semantics are as expressive as PBA, and with positive semantics as expressive as PCA.

**Theorem 8.** $\mathbb{L}^{>0}(\mathsf{PWA}) = \mathbb{L}^{>0}(\mathsf{PCA})$ *and* $\mathbb{L}^{=1}(\mathsf{PWA}) = \mathbb{L}^{=1}(\mathsf{PBA})$.

*Proof (sketch).* It suffices to show the first statement. The second then follows by duality, i.e., we can interpret a PBA$^{=1}$ $\mathcal{A}$ recognizing $L$ as a PCA$^{>0}$ recognizing $\overline{L}$ and just apply the construction to get a PWA$^{>0}$ $\mathcal{B}$ for $\overline{L}$, such that $\overline{\mathcal{B}}$ (with inverted accepting and rejecting states) is a PWA$^{=1}$ for $L$. In the first statement the $\subseteq$ inclusion is trivial, hence we only need to show that $\mathbb{L}^{>0}(\mathsf{PCA}) \subseteq \mathbb{L}^{>0}(\mathsf{PWA})$.

We construct a PWA$^{>0}$ consisting of two copies of the original PCA$^{>0}$, a *guess* copy and a *verify* copy. In the first copy, the automaton can guess that no final states will be visited anymore and switch to the verify copy, which is accepting, but where all transitions into final states are redirected to a rejecting sink. □

Next, we show that languages that can be accepted by both, a PWA with almost-sure semantics, and by a PWA with positive semantics, are regular and can be accepted by a DWA. For the proof, we rely on a characterization of DWA languages in terms of the Myhill-Nerode equivalence relation from [22]. So we first define this equivalence, and show that languages defined by PBA with positive semantics have only finitely many equivalence classes. Then we come back to the result for PWA.

For $L \subseteq \Sigma^\omega$, define the Myhill-Nerode equivalence relation $\sim_L \subseteq \Sigma^* \times \Sigma^*$ by $u \sim_L v$ iff $uw \in L \Leftrightarrow vw \in L$ for all $w \in \Sigma^\omega$. Then the following holds:

**Lemma 6 (Finitely many Myhill-Nerode classes).**
*Languages in* $\mathbb{L}^{>0}(\mathsf{PBA})$ *have finitely many Myhill-Nerode equivalence classes.*

*Proof.* Let $\mathcal{A} = (Q, \Sigma, \delta, \mu_0, F)$ be some PBA$^{>0}$ and $u \in \Sigma^*$ some word and let $\mu_u := \delta^*(\mu_0, u)$ be the probability distribution on states of $\mathcal{A}$ after reading $u$. Pick any $w \in \Sigma^\omega$ and notice that $uw \in L = L^{>0}(\mathcal{A})$ iff there exists some state $q$ such that $\mu_u(q) > 0$ and the probability to accept $w$ from $q$ is also $> 0$, as the product of two positive numbers clearly still is positive. But then, for any two $u, v \in \Sigma^*$ we have that whenever $\mu_u(q) > 0 \Leftrightarrow \mu_v(q) > 0$ for all $q$, then we have $uw \in L \Leftrightarrow vw \in L$ for all $w \in \Sigma^\omega$ by the reasoning above, as the exact value does not matter for acceptance, and therefore $u \sim_L v$. But as there are only at most $2^{|Q|}$ different possibilities how values in a distribution $\mu$ over $Q$ are either equal to or greater than 0, this is an upper bound on the number of different equivalence classes. □

**Theorem 9.** $\mathbb{L}^{>0}(\mathsf{PWA}) \cap \mathbb{L}^{=1}(\mathsf{PWA}) = \mathbb{L}(\mathsf{DWA}) = \mathbb{L}(\mathsf{PWA}^{0/1})$

*Proof.* The inclusions $\mathbb{L}(\mathsf{DWA}) \subseteq \mathbb{L}(\mathsf{PWA}^{0/1}) \subseteq \mathbb{L}^{>0}(\mathsf{PWA}) \cap \mathbb{L}^{=1}(\mathsf{PWA})$ are trivial, hence it remains to show $\mathbb{L}^{>0}(\mathsf{PWA}) \cap \mathbb{L}^{=1}(\mathsf{PWA}) \subseteq \mathbb{L}(\mathsf{DWA})$.

So let $L$ be a language from $\mathbb{L}^{>0}(\mathsf{PWA}) \cap \mathbb{L}^{=1}(\mathsf{PWA})$. We want to show that $L$ can be accepted by a DWA. We use the following characterization of DWA languages [22, Theorem 21]: The DWA languages are precisely the languages with finitely many Myhill-Nerode classes in the class $G_\delta \cap F_\sigma$ in the Borel hierarchy. The classes $G_\delta$ and $F_\sigma$ of the Borel hierarchy are often also referred to as $\Pi_2$ and $\Sigma_2$. We do not introduce the details of this hierarchy here, but rather refer the reader not familiar with these concepts to [22] and [8].

We already know that $L$ has finitely many Myhill-Nerode classes by Lemma 6 (as PWA are special cases of PBA). It remains to show that $L$ is in the class $G_\delta \cap F_\sigma$. It is known that PBA with almost-sure semantics define languages in $G_\delta$ [8, Lemma 3.2]. Hence $L$ is in $G_\delta$. Since $L$ is accepted by a PWA with positive semantics, the complement of $L$ is accepted by a PWA with almost-sure semantics (as noted at the beginning of this section). We obtain that the complement of $L$ is also in $G_\delta$ again by [8, Lemma 3.2]. This means that $L$ is in $F_\sigma$, which by definition consists of the complements of languages from $G_\delta$. □

Concluding this section, we show a result about weak automata with threshold semantics, which (not surprisingly) turn out to be even more expressive. A careful analysis of the PWA $\mathcal{A}$ in Fig. 2(a) shows the following result:

**Proposition 6.** *For all thresholds* $\lambda \in ]0, 1[$ *there exists a PWA* $\mathcal{A}$ *such that* $L^{>\lambda}(\mathcal{A})$ *is not regular and not PBA*$^{>0}$ *recognizable.*

Putting things together, we can say the following about threshold PWA, establishing the relation of $\mathbb{L}^{>\lambda}(\mathsf{PWA})$ to the other classes in Figure 3:

**Theorem 10 (Expressive power of threshold PWA).**

1. $\mathbb{L}^{>0}(\mathsf{PWA}) \subseteq \mathbb{L}^{>\lambda}(\mathsf{PWA}) \cap \mathbb{L}^{>0}(\mathsf{PBA})$.
2. $\mathbb{L}^{>\lambda}(\mathsf{PWA})$ *and* $\mathbb{L}^{>0}(\mathsf{PBA})$ *are incomparable (wrt. set inclusion).*
3. $\mathbb{L}^{>0}(\mathsf{PWA}) \subset \mathbb{L}^{>\lambda}(\mathsf{PWA}) \subset \mathbb{L}^{>\lambda}(\mathsf{PBA})$.

*Proof.* (1.) $\mathbb{L}^{>0}(\mathsf{PWA}) \subseteq \mathbb{L}^{>0}(\mathsf{PBA})$ by definition and $\mathbb{L}^{>0}(\mathsf{PWA}) \subseteq \mathbb{L}^{>\lambda}(\mathsf{PWA})$, as any PWA$^{>0}$ can be modified to a PWA$^{>\lambda}$ recognizing the same language by just adding an additional accepting sink and modifying the initial distribution, just as described in [4, Lemma 4.16] for general PBA.

(2.) By Proposition 6, there are languages recognized by PWA$^{>\lambda}$ that cannot be recognized with PBA$^{>0}$. To show that there are languages accepted by PBA$^{>0}$ that cannot be accepted by PWA$^{>\lambda}$ we can give a topological characterization of languages accepted by PWA by a simple adaptation of [8, Lemma 3.2] and combine it with other results shown in [8] to show that there are PBA$^{>0}$ that accept languages that cannot be accepted by PWA$^{>\lambda}$.

(3.) The first inclusion was discussed in (1.), the strictness follows from Proposition 6 and the fact that $\mathbb{L}^{>0}(\mathsf{PWA}) = \overline{\mathbb{L}^{=1}(\mathsf{PBA})} \subset \mathsf{BCl}(\mathbb{L}^{=1}(\mathsf{PBA})) = \mathbb{L}^{>0}(\mathsf{PBA})$, where the first equality is Theorem 8 and the second is shown in [8]. The second inclusion of the statement follows from (2.) and the fact from [4] that $\mathbb{L}^{>0}(\mathsf{PBA}) \subset \mathbb{L}^{>\lambda}(\mathsf{PBA})$. □

For the dual class $\mathbb{L}^{\geq\lambda}(\mathsf{PWA})$ one can show symmetric results that correspond to statements (1.) and (2.) above, for statement (3.) however there is no proof yet for the strictness of the inclusions (especially the second one), whereas the statement $\mathbb{L}^{=1}(\mathsf{PWA}) \subseteq \mathbb{L}^{\geq\lambda}(\mathsf{PWA}) \subseteq \mathbb{L}^{\geq\lambda}(\mathsf{PBA})$ is obvious. We leave this issue as an open question. Another interesting question is whether $> \lambda$ is equivalent to $< \lambda$ (or dually for $\geq / \leq$).

## 6 Conclusion

By using notions from ambiguity in classical Büchi automata, we were able to extend the set of easily (syntactically) checkable PBA which are regular under some or all of the usual semantics. As a consequence, ambiguity appears to be an even more interesting notion in the probabilistic setting, as here it in fact has consequences for the expressive power of automata, whereas in the classical setting there is no such effect. Our results also indicate that to get non-regularity, one requires the use of certain structural patterns which at least imply the existence of the ambiguity patterns that we used. It is an open question whether it is possible to identify more fine-grained syntactic characterizations, patterns or easily checkable properties which are just over-approximated by the ambiguity patterns and are required for non-regularity.
